# Bioresorbable Multilayer Photonic Cavities as Temporary Implants for Tether-Free Measurements of Regional Tissue Temperatures

**DOI:** 10.34133/2021/8653218

**Published:** 2021-01-15

**Authors:** Wubin Bai, Masahiro Irie, Zhonghe Liu, Haiwen Luan, Daniel Franklin, Khizar Nandoliya, Hexia Guo, Hao Zang, Yang Weng, Di Lu, Di Wu, Yixin Wu, Joseph Song, Mengdi Han, Enming Song, Yiyuan Yang, Xuexian Chen, Hangbo Zhao, Wei Lu, Giuditta Monti, Iwona Stepien, Irawati Kandela, Chad R. Haney, Changsheng Wu, Sang Min Won, Hanjun Ryu, Alina Rwei, Haixu Shen, Jihye Kim, Hong-Joon Yoon, Wei Ouyang, Yihan Liu, Emily Suen, Huang-yu Chen, Jerry Okina, Jushen Liang, Yonggang Huang, Guillermo A. Ameer, Weidong Zhou, John A. Rogers

**Affiliations:** ^1^Department of Materials Science and Engineering, Northwestern University, Evanston, Illinois 60208, USA; ^2^Querrey Simpson Institute for Bioelectronics, Northwestern University, Evanston, Illinois 60208, USA; ^3^Department of Electrical Engineering, University of Texas at Arlington, Arlington, TX 76019, USA; ^4^Department of Mechanical Engineering, Northwestern University, Evanston, Illinois 60208, USA; ^5^Department of Chemistry, Northwestern University, Evanston, Illinois 60208, USA; ^6^Department of Biomedical Engineering, Northwestern University, Evanston, Illinois 60208, USA; ^7^Department of Materials Science and Engineering, University of Illinois Urbana-Champaign, Urbana, Illinois 61801, USA; ^8^Academy for Advanced Interdisciplinary Studies, Peking University, Beijing, China; ^9^Department of Aerospace and Mechanical Engineering, University of Southern California, Los Angeles, CA 90089, USA; ^10^The Center for Developmental Therapeutics, Northwestern University, Evanston, Illinois 60208, USA; ^11^Center for Advanced Molecular Imaging, Northwestern University, Evanston, Illinois 60208, USA; ^12^Department of Electrical and Computer Engineering, Sungkyunkwan University, Suwon, Republic of Korea; ^13^School of Advanced Materials Science and Engineering, Sungkyunkwan University (SKKU), Suwon 16419, Republic of Korea; ^14^Department of Neurobiology, Northwestern University, Evanston, Illinois 60208, USA; ^15^Department of Chemical Engineering, Northwestern University, Evanston, Illinois 60208, USA; ^16^Department of Civil and Environmental Engineering, Northwestern University, Evanston, Illinois 60208, USA; ^17^Department of Electrical Engineering and Computer Science, Northwestern University, Evanston, Illinois 60208, USA; ^18^Northwestern Medicine, Feinberg School of Medicine, Northwestern University, Evanston, Illinois 60208, USA; ^19^Center for Advanced Regenerative Engineering, Northwestern University, Evanston, Illinois 60208, USA

## Abstract

*Objective and Impact Statement*. Real-time monitoring of the temperatures of regional tissue microenvironments can serve as the diagnostic basis for treating various health conditions and diseases. *Introduction*. Traditional thermal sensors allow measurements at surfaces or at near-surface regions of the skin or of certain body cavities. Evaluations at depth require implanted devices connected to external readout electronics via physical interfaces that lead to risks for infection and movement constraints for the patient. Also, surgical extraction procedures after a period of need can introduce additional risks and costs. *Methods*. Here, we report a wireless, bioresorbable class of temperature sensor that exploits multilayer photonic cavities, for continuous optical measurements of regional, deep-tissue microenvironments over a timeframe of interest followed by complete clearance via natural body processes. *Results*. The designs decouple the influence of detection angle from temperature on the reflection spectra, to enable high accuracy in sensing, as supported by in vitro experiments and optical simulations. Studies with devices implanted into subcutaneous tissues of both awake, freely moving and asleep animal models illustrate the applicability of this technology for in vivo measurements. *Conclusion*. The results demonstrate the use of bioresorbable materials in advanced photonic structures with unique capabilities in tracking of thermal signatures of tissue microenvironments, with potential relevance to human healthcare.

## 1. Introduction

The local temperatures of tissue microenvironments can serve as simple, yet important, diagnostic metrics relevant to a wide range of diseases and disorders [1-6], including those associated with chronic inflammation, traumatic injury, immunological irregularities, infections, and transplant rejection processes. Temperature is useful in these and other contexts because of the essential role that thermoregulatory processes play in maintaining normal cellular functions through a homeostatic balance between energy production and dissipation coordinated through metabolic mechanisms, local tissue perfusion, and hemodynamics [7-9]. Abnormalities in absolute values and/or temporal patterns of regional tissue temperatures can arise from certain immune responses and metabolic adjustments. These signatures can provide early signs of critical illness, to allow for proactive treatments and intervention [10]. Traditional thermal sensors based on infrared digital cameras, thermometers, thermistors, and resistive thermal detectors effectively support noninvasive measurements at the surfaces of the skin or of certain body cavities that are physically accessible [11-14]. Precise and continuous measurements of temperatures at regions deep inside the body, by contrast, require invasive probes and/or disruptive surgical interventions, with potential for adverse effects, including immune responses and pain/discomfort [15]. Furthermore, such strategies rely on permanent devices with wired connections for readout, when many scenarios demand wireless operation and/or temporary monitoring for time periods that match natural biological processes such as wound healing.

Bioresorbable (or equivalently bioabsorbable) electronic and optical technologies overcome these challenges through advanced sensing and stimulation capabilities that can be deployed in deep-tissue regions as temporary platforms, with minimal disruptions [16-19]. Constructed with materials that can undergo hydrolysis, enzymatic degradation, and/or oxidation in surrounding biofluids, these systems disappear in the body after a defined period of stable operation, thereby eliminating the necessity and associated risks of a secondary surgical extraction. Published examples of bioresorbable devices include (1) optical probes to monitor tissue oxygenation, neural activity, and cerebral temperature [20, 21]; (2) electronic sensors to measure pressures in the intracranial, intraocular, and intravascular spaces [22, 23]; and (3) wireless stimulators to provide a nonpharmacological means to accelerate healing of damaged peripheral nerves [24]. Optical technologies are in increasing use in modern medicine, with capabilities not only in monitoring physiological status and biochemical species but also in light-based therapies and treatments [25].

This paper presents materials, device architectures, physical and biochemical characteristics, and *in vivo* demonstrations of an implantable optical sensor for remote, tether-free measurement of deep-tissue temperature, in platforms that are entirely bioresorbable. The design exploits multilayers of silicon oxides (SiO_x_), silicon nitrides (SiN_y_), and silicon (Si), as temperature-modulated reflective photonic cavity structures designed with a spectroscopic response that depends on local temperature. A peak-detection algorithm identifies key features in the reflection spectra to allow robust measurements of temperature by comparing positions of resonances and their relative shifts to calibration standards, with computational modeling as a guide. System-level demonstrations using devices implanted into subcutaneous regions of both freely moving and asleep mice establish the feasibility and accuracy of the devices in monitoring temperature of tissue microenvironments buried underneath the skin. Studies of the dissolution of the constituent materials and their biodistribution through various internal organs highlight processes by which these optical devices undergo bioresorption.

## 2. Results and Discussion

**Figure 1:**
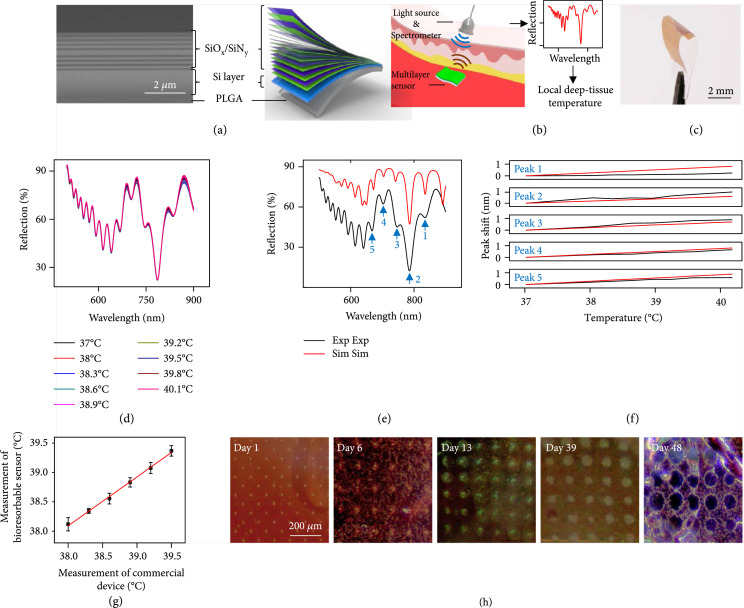
Bioresorbable multilayer photonic cavity structure for remote, wireless monitoring of temperature: (a) Left: cross-sectional SEM image of the multilayer structure. Right: schematic illustration of the compositional layout. The sensor consists of a cavity defined by a distributed Bragg reflector (DBR) (labeled SiO_x_/SiN_y_), a separate Fabry-Perot (F-P) cavity (labeled Si layer), and a polymeric substrate (labeled PLGA); (b) schematic illustration of a device based on this structure implanted into deep tissue. Tracking the positions of peaks in the measured reflection spectra enables measurements of changes in the temperature of local tissues; (c) image of a flexible device supported on a bioresorbable film of poly(lactic-co-glycolic acid) (PLGA); (d) reflection spectra measured in free space as a function of ambient temperature; (e) experimental and simulation results for the reflection spectrum of a multilayer photonic cavity; (f) experimental and simulation results of shifts in the positions of peaks in these spectra as a function of temperature; (g) temperatures determined from these spectra compared with those captured using a commercial thermometer (Neurolog, Inc.); (h) images of a device collected at several stages of dissolution in a solution of phosphate-buffered saline (PBS, pH=7.4) at room temperature. The periodic arrays of holes created during device fabrication for reliable transfer printing show a gradual increase in size during immersion in PBS.

Figure 1(a) presents the design of a bioresorbable multilayer cavity structure through a cross-sectional scanning electron microscope image and a schematic illustration. The system consists of three parts: (i) a distributed Bragg reflector (DBR) made of alternating layers of silicon oxides (SiO_x_) and silicon nitrides (SiN_y_) with a layer of SiO_x_ (twice the thickness of the others) in the center to yield a defect cavity. The dimensional details and the profiles of refractive index appear in Supplementary Figure 1. This DBR structure modulates the reflection spectrum across bands at 539 nm~581 nm, 634 nm~679 nm, and 825 nm~833 nm, with corresponding full widths at half maximum (FWHM) of 54 nm, 72 nm, and 46 nm, respectively (Supplementary Figure 2a); (ii) a 1.5 *μ*m thick membrane of monocrystalline silicon (Si), to create a Fabry-Perot resonance (Si F-P cavity) with reflection spectrum shown in Supplementary Figure 2b; and (iii) a 10 *μ*m thick substrate of poly(lactic-co-glycolic acid) (PLGA) to provide mechanical support during surgical implantation. The total thickness of the multilayer photonic cavity is ~13.6 *μ*m. The samples studied here have lateral dimensions of 3 mm by 3 mm.

**Figure 2:**
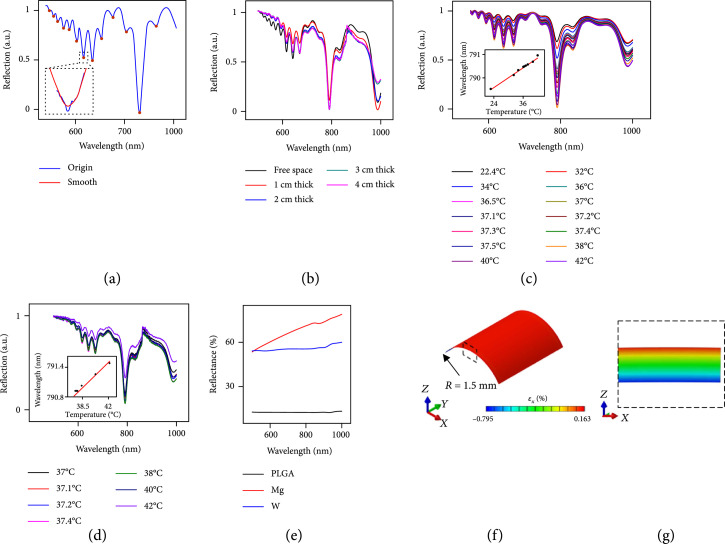
*In vitro* demonstrations of temperature sensing: (a) reflection spectra of a multilayer photonic cavity measured in free space and through pieces of raw chicken breast with thicknesses of 1 cm, 2 cm, 3 cm, and 5 cm; (b) reflection spectra of a device under a 1 cm thick piece of chicken tissue at various temperatures; (c) reflection spectra of a device under a 5 cm thick piece of chicken tissue at various temperatures; (d) a denoising algorithm determines the location of peaks for each measured spectrum. A flow chart that illustrates the algorithm appears in Supplementary Figure 3; (e) reflection spectra of a bare 10 *μ*m thick PLGA film, as coated with a 50 nm thick film of Mg, and a 50 nm thick film of W, respectively. The spectra show a significant increase of reflectivity by coating a bioresorbable metallic layer (W or Mg); (f) mechanical response of a bioresorbable multilayer photonic cavity structure to bending, as determined by finite element analysis. The results show the strain distribution of a device (length×width, 3 mm×3 mm) during bending to a radius of curvature of 1.5 mm. Supplementary Figure 7 shows the corresponding strains and stresses for each layer; (g) a zoom-in view of the strain distribution at a cross-sectional area (corresponding to the dashed square in (f)) of the multilayer photonic cavity structure. The fracture strain for both SiO_x_ and SiN_y_ is around 1%.

Figure 1(b) shows the operational concept where analysis of the reflection spectrum enables evaluation of temperature. Implantation of a multilayer photonic cavity sensor into a deep-tissue location allows the reflection spectrum to be captured by placing a fiber-based optical spectrometer onto the skin with an orientation approximately perpendicular to the plane of the cavity. Miniaturized dimensions and skin-comparable bending stiffnesses (~15 N∙m^-1^; Figure 1(c), Supplementary Figure 1d) with care in implantation procedures avoid excessive curvature that could distort the reflected spectra; these features also effectively minimize inflammation, scar formation, and tissue damage (additional details on the mechanics of the multilayer photonic cavity appear in Figure 2(g) and Supplementary Figure 7). Figure 1(d) shows the reflection spectra measured from this structure in free space at various temperatures. Changes in temperature cause spectral shifts of the resonant peaks due to the thermooptical effect of the constituent materials (dn/dT$\sim 2\times 10^{-4}$ K^-1^, $2\times 10^{-5}$ K^-1^, and $1\times 10^{-6}$ K^-1^, respectively, for Si, SiN_y_, and SiO_x_). Supplementary Figure 2c shows the refractive index of a Si micromembrane (1.5 *μ*m thick, supported on a 10 *μ*m thick PLGA film) as a function of temperature (between 22°C and 42°C) and wavelength (ranging from 500 nm to 900 nm).

The underlying mechanisms can be understood by analogy to a simple F-P cavity. Equation (1) is an expression of a resonant peak wavelength of the q^th^ order for such a cavity, where n is the refractive index, t is the thickness of the cavity, q is an integer order number, and $\lambda_{q}$ is the q^th^*-*order peak wavelength. Equation (2) defines the thermal expansion coefficient, α, where t is the thickness of the cavity [26]. Differentiating Equation (1) yields Equation (3), which reveals the dependence on the coefficient of thermal expansion and the thermooptic coefficient. Reported values for the former quantity for Si, SiO_2_, and Si_3_N_4_ are $2.6\times 10^{-6}$°C^-1^, $5.6\times 10^{-7}$°C^-1^, and $3.3\times 10^{-6}$°C^-1^, respectively [27-30]. The thermooptic coefficients of these same materials are $2\times 10^{-4}$ K^-1^, $2\times 10^{-5}$ K^-1^, and $1\times 10^{-6}$ K^-1^, respectively. The shifts in the resonant peaks for the structures reported here, therefore, can be considered to arise almost entirely from the thermooptic effect [27-30].

(1)λq=2ntq,(2)α=1tdtdT,(3)ddTλq=2qdndTt+dtdTn=2qdndTt+αtn=2tqdndT+αn≅2tqdndT=λqndndT.

Doped Si, including both p-type and n-type, has a refractive index that is approximately the same as that of undoped Si at visible wavelengths, with a decrease with increasing doping concentration in the near-infrared and infrared regimes [31, 32]. The thermooptic coefficient depends weakly on doping levels for both n-type and p-type Si [33]. For SiO_x_ and SiN_y_, increasing the concentration of oxygen or nitrogen decreases the refractive indexes [34, 35], while the thermal-optic coefficients are largely independent of stoichiometry [26]. The measured elemental ratios for SiO_x_ and SiN_y_ used in this work are Si 37% and O 63% and Si 53.8%, N 43.8%, and O 2.4%, respectively.

Figure 1(e) compares experimental measurements with simulation results for a reflection spectrum of the multilayer photonic cavity. These simulations (Figure 1(e)) rely on thin-film optic transfer matrix techniques implemented in MATLAB. Material dispersions originate from Palik and CRC databases [36, 37]. Fitting to experimental measurements involves a series of small adjustments to the thicknesses of the various material layers, in a manual process constrained to deviations that are no more than 5% of the nominal values. Within a range of temperatures relevant to biological systems (35°C to 45°C), linear changes in the refractive indices can be assumed to capture the temperature dependence of the full reflection spectra. Figure 1(f) shows the temperature dependence of peak positions (labeled 1, 2, 3, 4, and 5 in Figure 1(e)) in simulated spectra (labeled red line in Figure 1(f)) based on a thermooptic coefficient of $2\times 10^{-4}$ K^-1^ for Si. The results closely match experimental measurements (labeled black line in Figure 1(f)). Calibration relies on optical simulations to determine the positions of the reflection peaks as a function of temperature and angle of incident light. Figure 1(g) compares temperature measurements performed with a multilayer photonic cavity sensor calibrated in this way and a commercial thermal sensor (NTC thermistor). The measurement accuracy (standard deviation compared with the reference commercial sensor) is ~0.13°C, and the precision (standard error from 50 repeated cycles of measurements) is ~0.07°C (Figure 1(g)).

Besides their optical characteristics, as mentioned previously, a key unique feature of these systems is that all of the constituent materials dissolve by hydrolysis to biocompatible end products, as the basis for bioresorption. The reactions include (1) $\text{Si}+4{\text{H}}_{2}\text{O}\to \text{Si}{\left(\text{OH}\right)}_{4}+2{\text{H}}_{2}$; (2) $\text{Si}{\text{O}}_{2}+2{\text{H}}_{2}\text{O}\to \text{Si}{\left(\text{OH}\right)}_{4}$; and (3) ${\text{Si}}_{3}{\text{N}}_{4}+12{\text{H}}_{2}\text{O}\to 3\text{Si}{\left(\text{OH}\right)}_{4}+4\text{N}{\text{H}}_{3}$, to lead to slow dissolution in biofluids (Figure 1(h)) with experimentally observed rates consistent with previous reports on these materials [20, 21]. The result leads to a natural process of device clearance from the body with total masses of NH_3_ and Si(OH)_4_ generated from dissolution that are less than 3 *μ*g and 34.6 *μ*g, respectively. By comparison, the estimated daily intake of NH_3_ and Si(OH)_4_ is 1 mg and 35 mg, respectively [38-40].

### 2.1. Multilayer Photonic Cavity Designs

**Figure 3:**
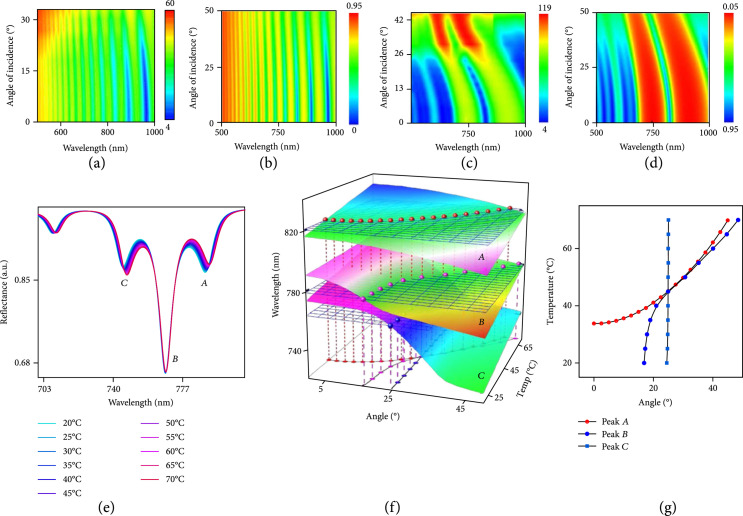
Optical properties of a bioresorbable multilayer photonic cavity structure and its sensing mechanism. Experimental measurements (a) and simulation results (b) of transmission spectra for a 1.5 *μ*m thick Si membrane at incidence angles between 0° and 32°. Experimental measurements (c) and simulation results (d) of transmission spectra for a SiN_x_/SiN_y_ multilayer structure at incidence angles between 0° and 32°. (e) Simulation results of a bioresorbable multilayer photonic cavity structure at temperatures between 20°C and 70°C. (f) 3D surface plots of peaks A, B, and C (labeled in (e)) at various temperatures and angle of incident light. (g) Each measurement yields a set of values for peaks A, B, and C. Applying these values to the 3D surface plots (shown in Figure 3(f)) yields intersection curves for peaks A, B, and C, respectively. The point of intersection defines the temperature and angle of incident light.

Both the Si F-P cavity and the DBR defect cavity (Figure 1(a)) modulate the spectrum of the reflected light in a way that depends not only on temperature but also on the angle of incident light (Supplementary Figures 3 and 4). Decoupling these two effects can eliminate uncertainties in the temperature measurement that could arise from variability in the angle. Previously reported classes of optically interrogated pressure sensors and related devices use physically coupled optical fibers to fix the angle [41], but with disadvantages that follow from the associated physical tether. Figures 3(a) and 3(b) show experimental and simulation results for reflection spectra of the Si F-P cavity (1.5 *μ*m thick) at various angles of incident light. The resonant peaks shift by ~0.3 nm per degree, with good agreement between experiment and simulation (Figures 3(a) and 3(b) and Supplementary Figure 2b). The DBR defect cavity exhibits a large angular dependence, with shifts of ~1.32 nm per degree, as shown in Figures 3(c) and 3(d). This cavity presents two major filtered bands (from 400 nm to 670 nm and from 1050 nm to 1200 nm) with a local reflectance minimum at a wavelength of ~860 nm (Supplementary Figure 4a). Increasing the incident angle (from 0^o^ to 35^o^) causes blueshifts in the reflectance features (peaks and dips) for both cavities. Increasing the temperature leads to redshifts.

Table 1 summarizes the temperature and angle sensitivity of the Si F-P and DBR defect cavities. The dramatic differences between these sensitivities provide the basis for determining the angle and the temperature separately from combined analysis of reflection spectra of both structures. Here, a multilayer structure that consists of an F-P cavity with monocrystalline Si and a DBR cavity with 8 bilayers of SiO_x_ and SiN_y_ and a defect layer of SiO_x_ yields a reflection spectrum that corresponds to the linear superposition of F-P resonances from the Si cavity and stops bands from the defect mode (around 780 nm) of the DBR cavity (Figures 1(d) and 3(e)). Compared to the DBR, the Si F-P cavity is more sensitive to changes in temperature but less sensitive to angle (Table 1). A critical consideration in this design is in thicknesses that place the wavelength of the defect peak in between those of peaks associated with the F-P resonances. This scheme effectively preserves the quality factors of individual resonances associated with the F-P and DBR cavities by minimizing their spectral overlap.

**Table 1:** Sensitivities of temperature and angle for the Si F-P cavity and DBR defect cavity.

	Si F-P cavity	DBR defect cavity
Temperature (nm/°C)	0.047	10^-15^
Incident angle (nm/°)	-0.30	-1.32

Sensing involves first constructing calibration surfaces defined by the temperature and angle dependence of the reflection peaks (Figure 3(f)) and then mapping the measured peaks onto these surfaces (Figure 3(g)). Mathematically, each calibration surface can be described with a fitting function (Figure 3(f)), such that measured peak values can be transformed into values of incident angle (θ) and temperature (T) (Figure 3(g)). In an ideal case, the temperature can be determined from only two peaks in the reflection spectrum: one for the DBR defect mode and one for an adjacent Si F-P mode. If the defect mode is approximately temperature independent over a range of 30° in incident angle, then its peak position defines the incident angle. Hence, the positions of the F-P peaks then determine the temperature at this incident angle (Figure 3(f)). In practical cases, the thicknesses of the layers deviate slightly from the design parameters, in a way that compromises the desired temperature independence of the position of the DBR defect mode. Here, mapping one defect peak and two F-P peaks onto the corresponding surfaces and projecting the intersection lines onto the temperature-angle (T-θ) plane lead to three routes that all pass around a single T-θ point as shown in Figures 3(e)-3(g). Increasing the number of F-P peaks in the T-θ maps (e.g., Figure 3(f)) can decrease the degree of uncertainty, thus further improving the precision of the measurement of temperature.

### 2.2. In Vitro Demonstrations of Sensing

Locating the spectral positions of resonant peaks defined with limited sampling resolution (typically, 0.01 nm~1 nm) relies on an algorithm that combines preprocessing and a zero-crossing filter applied to a linear interpolation of the spectral gradient around these peaks (Figure 2(a) and Supplementary Figure 5). Figure 2(b) shows reflection spectra from a multilayer photonic cavity (length×width, 1 cm×1 cm) buried underneath pieces of uncooked chicken breast tissue with various thicknesses. Increasing the thickness increases the scattering and absorption associated with optical interrogation. The measured spectra retain features sufficient for accurate extraction of peak positions even when probed underneath tissue with thickness of 4 cm (Figure 2(b)). Figures 2(c) and 2(d) show measurements performed through 1 cm and 3 cm thick tissue, respectively, at various temperatures. The experimental procedures appear in the method section, and the experimental setup for in vitro measurements appears in Supplementary Figure 13. The insets for Figures 2(c) and 2(d) show measured positions of a resonant peak (labeled “1” in Figure 1(e)) as a function of temperature. The results indicate a linear relationship with a slope of ~85.8 pm/°C and measurement accuracy of ~0.13°C. Tissue heterogeneity along the light path introduces additional light scattering and absorption features. These effects can modulate the resultant reflection spectrum of the photonic cavity embedded inside, thus further decreasing the sensitivity. As shown in Supplementary Figure 8, slices of muscle tissue from a bovine model (4 mm thick) with a low concentration of fat (10%) and a relatively high tissue homogeneity (compared with that of samples with 60% fat) show only modest disruption of the reflection spectrum, with key resonant peaks effectively preserved. For the samples with 35% fat, the peak quality is acceptable for thermal sensing with relatively consistent sensitivity compared with those of samples with 10% fat. For the samples with 60% fat, most peaks are still recognizable, while the FWHMs increase more than 9 nm compared with those of samples with 10% fat. These effects likely arise from the highly scattering characteristics associated with the inhomogeneous distribution of fat tissue. Moreover, the spectroscopic measurements use an external source (Tungsten Halogen Light Source, Ocean Insight) of white light with irradiance around 30 mW/cm^2^, with negligible heating of the device and surrounding tissue, as shown in Supplementary Figure 8b. Depositing a thin, bioresorbable reflective layer (such as magnesium (Mg) or tungsten (W)) onto the backside of a multilayer photonic cavity enhances the strength of the signal. Figure 2(e) shows reflection spectra of a PLGA film (thickness 10 *μ*m) and a PLGA film (thickness 10 *μ*m) with a 50 nm thick layer of Mg and with a 50 nm thick layer of W. The measurements on the metal-coated films show an enhanced reflection, from ~20% to >55% for W and to >65% for Mg, across a range of wavelengths from 500 nm to 1000 nm compared with that of an uncoated film (Figure 2(e)). The thickness and choice of the reflective coating layer (W or Mg, with dissolution rate around $1.7\times 10^{-3}$ *μ*m/h and 0.07 *μ*m/h, respectively [42]) determine the functional time to serve as a reflective interface during its immersion in PBS. Another scheme relies on patterning the film with arrays of pyramidal features (length of side, 25 *μ*m; depth, 15 *μ*m) to form microscale retroreflectors that can further enhance the reflection signals (Supplementary Figure 7). Supplementary Figure 7b compares reflection spectra between PLGA with and without microretroreflectors, indicating a dramatic increase (from ~20% to ~60%). Incorporating this retroreflective design into the multilayer sensor enhances the reflected spectral signal with negligible changes in the peak profiles (Supplementary Figure 7c).

The mechanical bendability of these structures is important in minimizing irritation at their interfaces with soft, moving tissues in live animal models. Figures 2(f) and 2(g) show the distribution of strain throughout a sensor (length×width, 3 mm×3 mm) during bending to a radius of curvature of 1.5 mm. The maximum strains in the SiO_x_, SiN_y_, Si, and PLGA layers are 0.16%, 0.16%, 0.10%, and 0.80%, respectively. Each of these values lies beneath the fracture threshold of the corresponding material. Supplementary Figure 8 shows similar results for a geometry where the SiO_x_-SiN_y_ DBR defect cavity is under tensile strain, and the Si F-P cavity and PLGA layer are under compressive strain. In the DRB defect cavity, the strain increases as the corresponding layers locate closer to the surface of the sensor (Supplementary Figure 8d), as expected based on elementary bending mechanics.

### 2.3. Studies of Dissolution and Bioresorption

**Figure 4:**
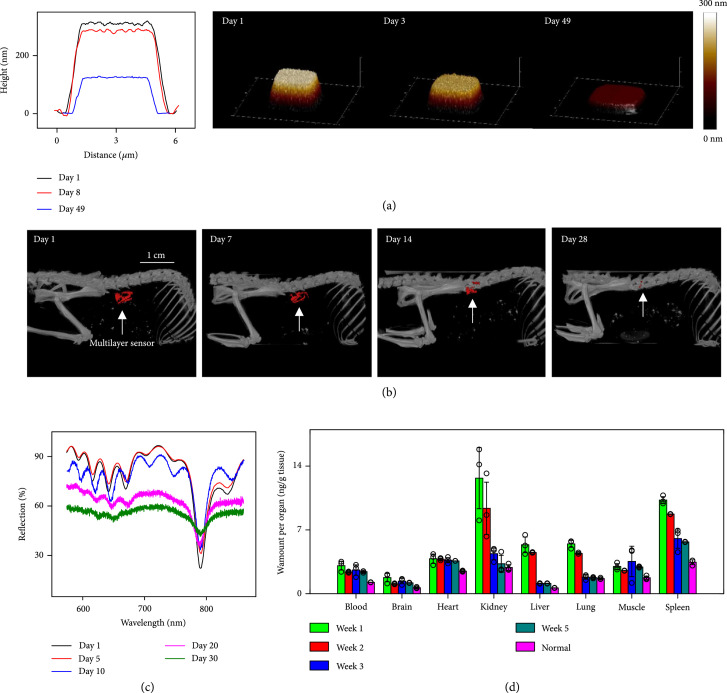
Bioresorption characteristics: (a) height profiles and AFM topographical images of a multilayer photonic cavity patterned into a square pad with sloping sidewalls, measured at various times after immersion in phosphate-buffered saline (PBS) (pH=7.4) at 37°C; (b) 3D-rendered computed tomography (CT) images of mice collected over 2 weeks after implantation of a device, showing gradual disappearance of W coatings on the bioresorbable photonic cavity, as an approximate representation of the bioresorption of the device; (c) measured reflection spectra of a multilayer photonic cavity during its immersion in PBS solution (pH=7.4) at 37°C; (d) *in vivo* biodistribution of tungsten (W) coatings from a bioresorbable photonic cavity. Here, the device consists of a multilayer photonic cavity structure (length×width, 3 mm×3 mm) supported on a 10 *μ*m thick PLGA film. Implantation was in the subcutaneous region near the flank region (n=12 biologically independent mice), with comparisons to control animals (n=3 biologically independent mice). Euthanizing three experimental mice (n=3 biologically independent mice) at weeks 1, 2, 3, and 5 enabled analysis of biodistribution of implanted bioresorbable photonic cavities. Inductively coupled plasma mass spectrometry (ICP-MS) defines the concentrations of W, in blood and organs (brain, heart, kidney, liver, lung, muscle, and spleen) explanted at 1, 2, 3, and 5 weeks after implantation.

As mentioned previously, a unique feature of the multilayer systems reported here is that all of the constituent materials dissolve completely in simulated and actual biofluids into biocompatible end products that can be cleared through natural metabolic processes. Patterning alternating layers of SiO_x_ (thickness 139 nm) and SiN_y_ (thickness 102 nm) into microscale pads (length×width, 5.5 μm×5.5 μm) facilitates the use of atomic force microscopy (AFM) to characterize the total thicknesses at various stages of immersion in phosphate-buffered saline (PBS) at 37°C (pH=7.4) (Figure 4(a)). Hydrolysis of these layers leads to dissolution rates of 6±4 nm/day for SiO_x_ and 15±5 nm/day for SiN_y_ (Figure 4(a)), consistent with previous reports on these materials in other contexts [20, 43]. Figure 4(b) and Supplementary Figure 9 show results for *in vivo* bioresoprtion of a multilayer photonic cavity (length×width×thickness, 3 mm×3 mm×18.57 *μ*m, consisting of a 2.07 *μ*m thick DBR, a 1.5 *μ*m thick Si membrane, a 10 *μ*m thick PLGA substrate, and two 200 nm thick layers of sputtered tungsten (W) coated on both sides to facilitate imaging) implanted at a subcutaneous region near the thigh of a mouse model. Images (Figure 4(b) and Supplementary Figure 9) obtained by computed tomography (CT) indicate gradual dissolution with full bioresorption of the W coatings at ~15 days after implantation. The W dissolves through a hydrolysis reaction, $2\text{W}+2{\text{H}}_{2}\text{O}+3{\text{O}}_{2}\to 2{\text{H}}_{2}\text{W}{\text{O}}_{4}$. Figure 4(c) shows the change in the reflection spectra of the multilayer photonic cavity during its immersion into PBS solution at 37°C (pH=7.4). For the initial 5 days of immersion, the resonant peaks show negligible drift and the FWHM of these peaks changes by less than 5 nm, thus effectively maintaining the measurement accuracy. After 5 days, the reflection spectra begin to change in a significant way. On day 20, peaks corresponding to the Si F-P cavity become unrecognizable. On day 30, most peaks are no longer observable (Figure 1(h)). Strategies to extend the functional lifetimes of the devices of this type include those that rely on nanomembranes of thermally grown silicon dioxide as encapsulation layers with low water vapor transmission rate (~10^-7^ g∙m^-2^∙d^-1^) and low dissolution rate (0.11 nm/day) [22, 44].

Figure 4(d) shows concentrations of W in the blood, brain, heart, kidney, liver, lung, muscle, and spleen tissues explanted from mice at 1, 2, 3, and 5 weeks after implantation, measured by inductively coupled plasma mass spectrometry (ICP-MS). The results indicate no abnormal accumulation of W for the 5-week implantation period, compared with those in the control group. Elevated concentrations during the first 2 weeks appear in the kidney, followed by a gradual decrease to a normal level, indicating renal clearance to maintain the metabolic balance of W inside the body. The spleen also shows an initial elevation in W concentration, followed by a gradual decrease to normal levels, indicating its immunological roles in metabolizing W. Biodistribution analysis of dissolution of Si micromembranes also indicate no adverse effects [20]. In vitro studies using a resazurin assay on cell metabolism and viability for the case of fibroblast cells from the human colon grown on device structures suggest that the dissolved components are biocompatible without significant cytotoxicity effects. (Supplementary Figure 10).

### 2.4. Temperature Sensing in Awake Animal Models

**Figure 5:**
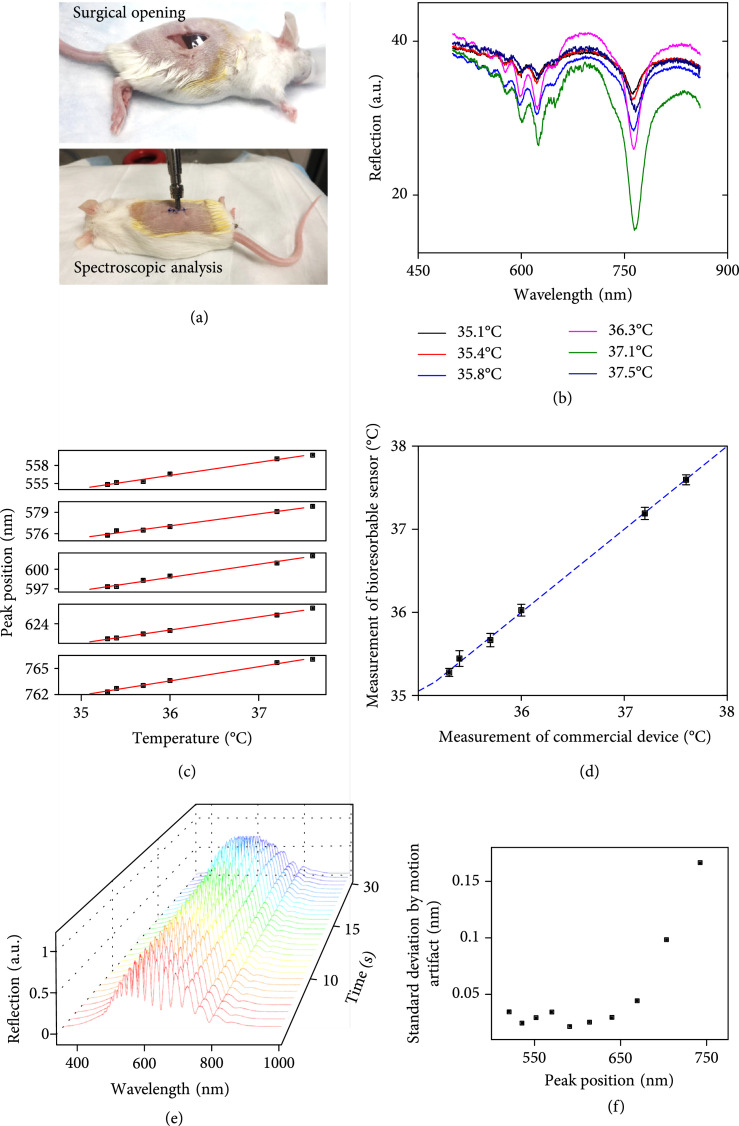
Evaluations in live animal models: (a) upper: image of a bioresorbable multilayer photonic cavity structure implanted inside a surgically opened pocket at a subcutaneous region of a mouse. Lower: image of the surgical suture to close the pocket with the device implanted; (b) reflection spectra obtained using a fiber-coupled spectrometer pressed against the skin above the device. Placing the mouse onto a heating blanket increased the body temperature, as captured with the device and with a commercial sensor (Neurolog, Inc.); (c) measured positions of resonant peaks as a function of temperature. The plot indicates a linear relation between these peaks and the temperature; (d) calibrated temperature measured by the bioresorbable device compared with those obtained using a commercial thermometer; (e) reflection spectra of a bioresorbable multilayer photonic cavity collected in real-time while implanted in the subcutaneous region of an awake mouse housed at room temperature (23°C); (f) calculated standard deviation of peak positions extracted from these spectra. The measurements of resonant peaks at short-wavelength regime (from 500 nm to 600 nm) show a lower standard deviation induced by motion artifact, compared with those at long-wavelength regime (from 600 nm to 800 nm).

**Figure 6:**
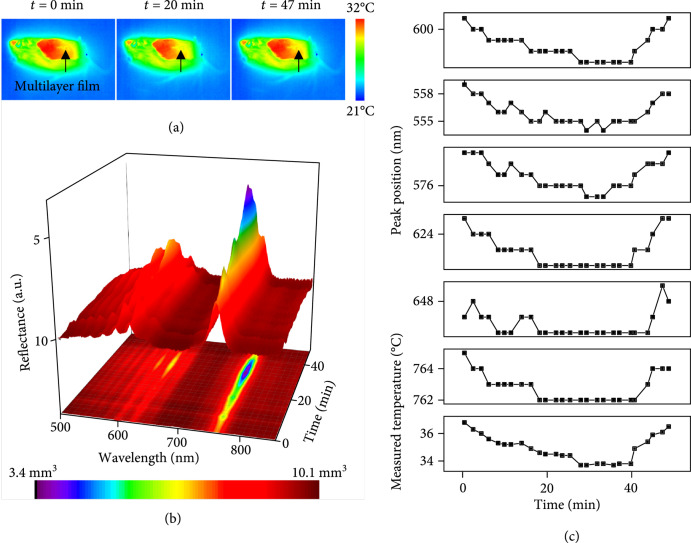
*In vivo* monitoring during sleep: (a) thermal images captured at various sleep stages of a mouse with a multilayer cavity implanted in a subcutaneous region near the thigh; (b) 3D surface plot of reflection spectra; (c) extracted positions of peaks of the reflection spectra and calibrated temperature at the implantation site as a function of time during sleep.

The use of these bioresorbable multilayer photonic cavity in live animal models (Figures 5 and 6 and Supplementary Figure 11) demonstrates their ability to sense temperature at targeted, local tissue microenvironments at subdermal regions in awake mouse models. Implantation of the devices into surgically opened pockets at subcutaneous regions near the thigh of mouse models followed by surgical closure using bioresorbable sutures (Figure 5(a)) allows external collection of reflection spectra using a fiber-coupled spectrometer through a shaved region of the skin (Supplementary Figure 11). Figure 5(b) shows representative spectra captured in this manner. Body movements as well as light scattering and absorption in the tissue and the fur contribute to the noise in the measurements [45]. Nevertheless, the algorithm described previously (Supplementary Figure 5) can accurately determine the positions of peaks in the spectra. The results show a linear relationship with temperature measured by a probe-based commercial sensor placed close to the surgical pocket (Figure 5(c)). Fitting each measured spectrum with positions of peaks from both the DBR and F-P cavities based on corresponding calibration curves (Figure 5(c)) yields temperature values that agree well with measurements using the commercial sensor (Figure 5(d)). The measurement accuracy is ~0.2°C and the precision is ~0.1°C. (Figure 5(d)). Small motions of the mouse, including respiration, muscle movements, and tissue deformation, can potentially affect the reflection spectral of the implanted photonic cavity sensor, thus decreasing the sensitivity. Figure 5(e) and Supplementary video 1 show real-time collection of reflection spectra collected from an awake mouse housed at room temperature (23°C). The spectra show that respiration of the mouse induces strong variations in the signal intensity which can be further utilized to derive respiration rate, while the variations in the position of resonant peaks are relatively small, as shown in Figure 5(f). The motion-induced standard deviation of resonant peaks at short-wavelength regime (from 500 nm to 600 nm) is below 0.05 nm, which is lower than that at long-wavelength regime (from 600 nm to 800 nm), around 0.1 nm. A combined analysis based of the full collection of resonant peaks can further minimize the effects of motion on the sensor sensitivity.

Measurements of temperature as a function of time during sleep provides a practical example of the use of this technology (Figure 6). Figure 6(a) shows a series of thermal images of a sleeping mouse with an implant as described above (Supplementary Figure 11). Continuous collection of reflection spectra provides information on peak positions as a function of time. Analysis according to previously outlined procedures defines the temperature of the implanted region for various stages of sleep (Figure 6(b)). Figure 6(c) shows the positions of reflection peaks and calibrated temperatures as a function of time beginning with initial asleep (t=0 min) and continuing until wakening (*t*~47 min). The measurements (Figure 6) show a slow decrease of core body temperature from 36.8°C to 33.7°C during the initial 27 mins of sleep, followed by a constant value of ~34°C, and then by an increase from 33.9°C to 36.5°C during the last 8 mins of sleep. These trends agree well with measurements using an infrared camera (FLIR Systems, Inc.) and are consistent with observations reported previously [46, 47].

## 3. Conclusion

The bioresorbable multilayer photonic crystal device introduced here represents a wireless temperature sensing platform that relies on *in vivo* spectroscopic measurements. A unique resonant cavity design decouples effects of the sensing parameter (temperature) from other interfering effects, including angle of incident light, tissue scattering, and absorption. The small size and compliant mechanics of the system minimize adverse effects such as inflammation and immune responses during and after implantation. The bioresorbability of all the constituent materials (Si, SiO_x_, SiN_y_, and PLGA) enables complete clearance of the implanted device after a certain period of stable operation. Strategies to establish this time period include the use of (i) conformal coatings of SiO_2_ with a dissolution rate, around 14 nm/day, that can prevent biofluids from contacting the multilayer photonic cavity during the encapsulation period [43], or (ii) conformal coatings of polyanhydride-based polymers (such as polybutanedithiol 1,3,5-triallyl-1,3,5-triazine-2,4,6(1H,3H,5H)-trione pentanoic anhydride) with controlled thickness, molecular weight, and degree of polymerization. The hydrophobicity and the surface-erosion characteristics of this polymer can effectively prevent permeation of biofluids, therefore ensuring the stable operation of devices encapsulated inside [48]. Studies of biodistribution of tungsten and bioresorption of Si, SiO_x_, and SiN_y_ during device implantation reveal no measurable toxic effects or accumulation of the implanted materials. *In vivo* monitoring of subcutaneous temperature in awake and asleep mice highlights potential applicability in biomedical research and clinical diagnosis at deep tissue. This concept of wireless optical sensing with bioresorbable photonic structures suggests additional unique opportunities in bio-optical materials and technologies in biomedical device design. The potential applications range from use as research tools in fundamental studies of the pathophysiology of critical diseases (such as cancer metastasis and neurological disorders) to possible utilization in guiding surgical procedures and monitoring recovery/rehabilitation from certain types of illness or injury.

## 4. Materials and Methods

### 4.1. Fabrication of Bioresorbable Multilayer Photonic Cavities

As shown in Supplementary Figure 12, fabrication began with programmed deposition of alternating layers of SiO_x_ and SiN_y_ using plasma-enhanced chemical vapor deposition (PECVD) onto a silicon-on-insulator wafer (thickness of device layer: 1500 nm; thickness of buried oxide layer: 1000 nm; base silicon (Si) layer polished to a thickness of 200 *μ*m). Spin coating polyimide (PI) at 3000 rpm for 30 s and baking at 250°C for 1 h formed a 2 *μ*m thick PI film on the surface of the multilayer. Etching under a vapor of xenon difluoride removed the Si wafer from the backside. Drop casting PLGA from an ethyl acetate solution (7 wt%) and baking at 70°C for 10 min produced a 10 *μ*m thick coating of PLGA on the multilayer to complete the fabrication. Supplementary Figure 13 shows experimental setup for optical characterization of the multilayer photonic cavity sensors.

### 4.2. Animal Model Studies

All procedures followed recommendations in the Guide for the Care and Use of Laboratory Animals of the National Institutes of Health. The Institutional Animal Care and Use Committee (IACUC) at Northwestern University (protocol IS00005877) approved the protocol. Female mice (CD1, age at initiation of the treatment: at least 6 weeks, but not more than 15 weeks, purchased from Charles River Laboratories) were acclimated up to 5 d before surgery. 2% isoflurane gas anesthetized animals during the implantation surgery. Following surgical exposure of a pocket in a subcutaneous region near the thigh (Supplementary Figure 9), implantation involves insertion of a square-shape multilayer photonic cavity coated with W (length×width×thickness, 3 mm×3 mm×30 *μ*m, thickness of the W coating layer, 50 nm) and bonding to the tissue with a bio-adhesive (3 M Vetbond tissue adhesive). Bioabsorbable suturing and gluing closed the surgically exposed region and completed the surgery. Mice were allowed to recover for 30 min prior to measurements.

### 4.3. Evaluation of Elemental Biodistribution, Hematology, and Blood Chemistry

Overnight exposure to ultraviolet radiation sterilized multilayer photonic cavities coated with W (length×width×thickness, 1 cm×1 cm×30 *μ*m, thickness of the W coating layer, 50 nm). The implantation procedures involved anesthetizing a female CD-1 mouse (Charles River, USA) with isoflurane gas (~2%), opening a 1 cm length pocket at the subcutaneous region near the thigh, inserting the device into the pocket, and suturing to close the surgical opening, as approved by the Institutional Animal Care and Use Committee (IACUC) of Northwestern University (Protocol IS00005877). Daily checking, weighing, and caring of the mice ensured their healthy condition and normal stress. Euthanization of 3 mice at weeks 1, 2, 3, and 5 after device implantation enabled extraction of blood and explantation and weighing of organs including the brain, heart, kidney, liver, lung, muscle, and spleen. Storing the organs in preweighed 15 mL conical metal-free tubes in -20°C fridge prepared tissue samples for biodistribution studies. Dissolving the tissues by adding 1.5 mL nitric acid and 0.35 mL hydrogen peroxide to each tube, holding the tubes in a water bath at 65°C for 5 h, diluting the dissolved tissue solutions 1 : 20 by adding Milli-Q water (MilliporeSigma, USA), and analyzing the samples by inductively coupled plasma mass spectrometry (ICP-MS) yielded the concentrations of W in the tissues obtained at 1, 3, 5, and 7 weeks after implantation.

### 4.4. Optical Simulations

The optical simulations used the Stanford Stratified Structure Solver, a frequency domain code to solve the linear Maxwell equations in layered periodic structures using the Rigorous Coupled Wave Analysis (RCWA) method and an S-matrix algorithm. The simulation began by constructing the multilayer photonic cavity structure in 3-dimensional Cartesian coordinates, followed by defining each layer with physical parameters including permittivity (both real part and imaginary parts) and thickness, consistent with experimental measurements and previous reports [49-51]. Defining parameters of incident light including various incident angles, intensities, polarization states, and wavelengths allowed computation of the intensity of the reflected light as a function of light incident angle and wavelength.

### 4.5. Algorithms for Locating Peaks in Measured Spectra

A zero-phase second-order Butterworth low-pass filter was used to suppress noise, with a cutoff coefficient of 5 normalized by the spectral resolution (fs). The local minima were found with minimum peak-to-peak distance of 5×fs. Each true peak was then estimated as a zero-crossing point on a gradient of a spectrum linearly interpolated from samples within fs from a detected discrete peak.

### 4.6. Finite Element Analysis (FEA)

The 3D FEA used the software suite Abaqus FEA to analyze the bending behaviors of bioresorbable multilayer photonic cavity structure. Four-node composite shell elements were used for the multilayer structure. The bending load was prescribed at two opposite edges without confining lateral deformation. The deformed 3D shape and stress/strain distributions at different locations for the multilayer structure can be obtained. In the simulation, silicon oxides (SiO_x_), silicon nitrides (SiN_y_), silicon, and PLGA are modelled as linear elastic materials, with Young’s moduli and Poisson’s ratios of $E_{\text{SiOx}}=66.3$ GPa, $\nu_{\text{SiOx}}=0.15$ for silicon oxides; $E_{\text{SiNy}}=166\;$GPa, $\nu_{\text{SiNy}}=0.23$ for silicon nitrides; $E_{\text{Si}}=130$ GPa, $\nu_{\text{Si}}=0.2$7 for silicon; and $E_{\text{PLGA}}=1.37$ GPa, $\nu_{\text{PLGA}}=0.44$ for PLGA.

## Data Availability

The main data supporting the results of this study are available within the paper and its Supplementary Information files. The raw and analysed datasets generated during the study are available for research purposes from the corresponding author on reasonable request.

## Supplementary Materials

Supplementary 1 Supplementary Figure 1: (a) 3D schematic illustration of a bioresorbable multilayer photonic cavity. (b) 2D schematic illustration of the device with the corresponding thicknesses for each layer shown on the right. (c) Depth profile of the refractive index for the distributed Bragg reflector (DBR) of alternating layers of silicon oxides (SiO_x_) and silicon nitrides (SiN_y_) with one double-thickness SiO2 layer in the center to serve as the DBR defect cavity. The refractive indices of SiO_x_ and SiN_y_ are 2.03 and 1.48, respectively. (d) Measured stiffness of a multilayer photonic cavity as a function of oscillation frequency. (e) Measured stress-strain curve during bending a multilayer device. Supplementary Figure 2: (a) experimental measurements and simulation results of transmission spectra of the SiO_x_/SiN_y_ multilayer layer at a 0° incidence angle. (b) Experimental measurements and simulation results for transmission spectra of a 1.5 *μ*m thick Si membrane at a 0° incidence angle. (c) Experimental measurements of refractive index of a Si membrane as a function of temperature (from 21°C to 50°C) and wavelength (from 500 nm to 1000 nm), determined using an ellipsometer. The device consists of a 1.5 *μ*m thick Si membrane supported on a 10 *μ*m thick PLGA film. Supplementary Figure 3: (a) measured reflection spectra, at 0° incidence angle, of a bioresorbable multilayer photonic cavity in free space as a function of environmental temperature. (b) Measured positions of the resonant peaks as a function of temperature. The plot indicates a linear relation between these positions and temperature. Supplementary Figure 4: (a) measured reflection spectra at 17° incidence angle for a bioresorbable multilayer photonic cavity in free space as a function of temperature. (b) Measured positions of resonant peaks as a function of temperature. The plot indicates a linear relation between these positions and temperature. Supplementary Figure 5: a flow chart for the denoising algorithm for locating each resonant peak of a measured spectrum. Supplementary Figure 6: (a) measured reflection spectra of a bioresorbable multilayer photonic cavity buried underneath 4 mm thick tissue slices from a bovine model with various compositions of fat, ranging from 10% to 60%, with the rest of the tissue slices mostly composed of muscle tissue. Supplementary Figure 7: (a) optical image and 3D optical image of a 10 *μ*m thick PLGA film with a bottom surface structured with arrays of microscale pyramidal features. (b) Reflection spectra of 10 *μ*m thick PLGA films with a flat surface and with a bottom surface structured with arrays of microscale corner cubes. (c) Reflection spectra of devices with and without retroreflective designs for the PLGA substrates. The devices are embedded in a 5 mm thick piece of chicken tissue at room temperature (~23°C). Supplementary Figure 8: finite element simulation on the mechanical response of a bioresorbable multilayer photonic cavity structure to bending. Supplementary Figure 9: computed tomography (CT) images (3D-rendered, coronal, axial, and sagittal views) of mice collected over 2 weeks after implantation of a bioresorbable multilayer photonic cavity, showing gradual disappearance of the device (highlighted by red circle). Supplementary Figure 10: cell viability calculated as the fraction of total living cells after conditioning the culture for 24 h. Supplementary Figure 11: images of the surgical procedure for implanting a bioresorbable multilayer photonic cavity. Supplementary Figure 12: 3D schematic illustration of key fabrication steps for a bioresorbable multilayer photonic cavity. Supplementary Figure 13: (a) experimental setup for optical characterization of the multilayer photonic cavity sensors embedded in an apple slice, in chicken breast tissue, and in phosphate-buffered saline. (b) Schematic illustration of the experimental setup.
