# Supplementary material for: Bioresorbable Multilayer Photonic Cavities as Temporary Implants for Tether-Free Measurements of Regional Tissue Temperatures
Source: BME Front. 2021 Jan 15;2021:8653218. doi: 10.34133/2021/8653218 (PMC10521677; doi:10.34133/2021/8653218)
Supplement: Supplementary 1 — Supplementary Figure 1: (a) 3D schematic illustration of a bioresorbable multilayer photonic cavity. (b) 2D schematic illustration of the device with the corresponding thicknesses for each layer shown on the right. (c) Depth profile of the refractive index for the distributed Bragg reflector (DBR) of alternating layers of silicon oxides (SiOx) and silicon nitrides (SiNy) with one double-thickness SiO2 layer in the center to serve as the DBR defect cavity. The refractive indices of SiOx and SiNy are 2.03 and 1.48, respectively. (d) Measured stiffness of a multilayer photonic cavity as a function of oscillation frequency. (e) Measured stress-strain curve during bending a multilayer device. Supplementary Figure 2: (a) experimental measurements and simulation results of transmission spectra of the SiOx/SiNy multilayer layer at a 0° incidence angle. (b) Experimental measurements and simulation results for transmission spectra of a 1.5 μm thick Si membrane at a 0° incidence angle. (c) Experimental measurements of refractive index of a Si membrane as a function of temperature (from 21°C to 50°C) and wavelength (from 500 nm to 1000 nm), determined using an ellipsometer. The device consists of a 1.5 μm thick Si membrane supported on a 10 μm thick PLGA film. Supplementary Figure 3: (a) measured reflection spectra, at 0° incidence angle, of a bioresorbable multilayer photonic cavity in free space as a function of environmental temperature. (b) Measured positions of the resonant peaks as a function of temperature. The plot indicates a linear relation between these positions and temperature. Supplementary Figure 4: (a) measured reflection spectra at 17° incidence angle for a bioresorbable multilayer photonic cavity in free space as a function of temperature. (b) Measured positions of resonant peaks as a function of temperature. The plot indicates a linear relation between these positions and temperature. Supplementary Figure 5: a flow chart for the denoising algorithm for locati [file 8653218.f1.docx]

**Supplementary Materials**

**Bioresorbable Multilayer Photonic Cavities as Temporary Implants for Tether-free Measurements of Regional Tissue Temperatures**

Wubin Bai,^1,2,†^ Masahiro Irie,^1,2,†^ Zhonghe Liu,^3,†^ Haiwen Luan,^2,14^ Daniel Franklin,^2^ Khizar Nandoliya,^4^ Hexia Guo,^1,2^ Hao Zang,^1^ Yang Weng,^1^ Di Lu,^2^ Di Wu,^5^ Yixin Wu,^1,2^ Joseph Song,^5^ Mengdi Han,^2^ Enming Song,^2,6^ Yiyuan Yang,^1,2^ Xuexian Chen,^2,7^ Hangbo Zhao,^2,8^ Wei Lu,^2^ Giuditta Monti,^5^ Iwona Stepien,^9^ Irawati Kandela,^2^ Chad R. Haney,^5,10^ Changsheng Wu,^2^ Sang Min Won,^11^ Hanjun Ryu,^2^ Alina Rwei,^2^ Haixu Shen,^1,2^ Jihye Kim,^2,17^ Hong Joon Yoon,^2,17^ Wei Ouyang,^2^ Yihan Liu,^1^ Emily Suen,^15^ Huang-yu Chen,^2^ Jerry Okina,^16^ Jushen Liang,^16^ Yonggang Huang,^1,2,14,18^ Guillermo A. Ameer,^5,12,19^ Weidong Zhou,^3^ John A. Rogers^1,2,4,5,12,13,14,19*^

^1^ Department of Materials Science and Engineering, Northwestern University, Evanston, Illinois 60208, USA.

^2^ Querrey Simpson Institute for Bioelectronics, Northwestern University, Evanston, Illinois 60208, USA.

^3^ Department of Electrical Engineering, University of Texas at Arlington, Arlington, TX 76019, USA.

^4^ Department of Chemistry, Northwestern University, Evanston, Illinois 60208, USA.

^5^ Department of Biomedical Engineering, Northwestern University, Evanston, Illinois 60208, USA.

^6^ Department of Materials Science and Engineering, University of Illinois Urbana-Champaign, Urbana, Illinois 61801, USA.

^7^ Academy for Advanced Interdisciplinary Studies, Peking University, Beijing, China.

^8^ Department of Aerospace and Mechanical Engineering, University of Southern California, Los Angeles, CA 90089, USA

^9^ The Center for Developmental Therapeutics, Northwestern University, Evanston, Illinois 60208, USA.

^10^ Center for Advanced Molecular Imaging, Northwestern University, Evanston, Illinois 60208, USA.

^11^ Department of Electrical and Computer Engineering, Sungkyunkwan University, Suwon, South Korea

^12^ Northwestern Medicine, Feinberg School of Medicine, Northwestern University, Evanston, Illinois 60208, USA.

^13^ Department of Electrical Engineering and Computer Science, Northwestern University, Evanston, Illinois 60208, USA.

^14^ Department of Mechanical Engineering, Northwestern University, Evanston, Illinois 60208, USA.

^15^ Department of Neurobiology, Northwestern University, Evanston, Illinois 60208, USA.

^16^ Department of Chemical Engineering, Northwestern University, Evanston, Illinois 60208, USA.

^17^ School of Advanced Materials Science and Engineering, Sungkyunkwan University (SKKU), Suwon 16419, Republic of Korea

^18^ Department of Civil and Environmental Engineering, Northwestern University, Evanston, Illinois 60208, USA.

^19^ Center for Advanced Regenerative Engineering, Northwestern University, Evanston, Illinois 60208, USA.

* Corresponding authors: John A. Rogers; [jrogers@northwestern.edu](mailto:jrogers@northwestern.edu)

**
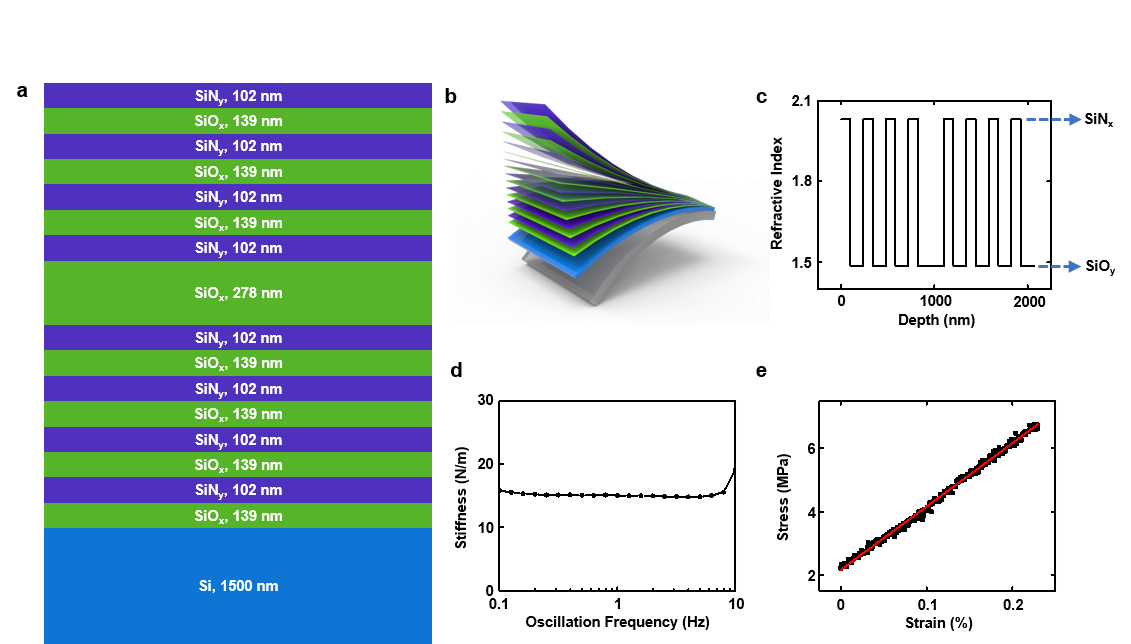
**

**Supplementary Figure 1.** **(a)** 3D schematic illustration of a bioresorbable multilayer photonic cavity. **(b)** 2D schematic illustration of the device with the corresponding thicknesses for each layer shown on the right. **(c)** Depth profile of the refractive index for the distributed Bragg reflector (DBR) of alternating layers of silicon oxides (SiO_x_) and silicon nitrides (SiN_y_) with one double thickness SiO_2_ layer in the center to serve as the DBR defect cavity. The refractive indices of SiO_x_ and SiN_y_ are 2.03 and 1.48, respectively. **(d)** Measured stiffness of a multilayer photonic cavity as a function of oscillation frequency. **(e)** Measured stress-strain curve during bending a multilayer device.


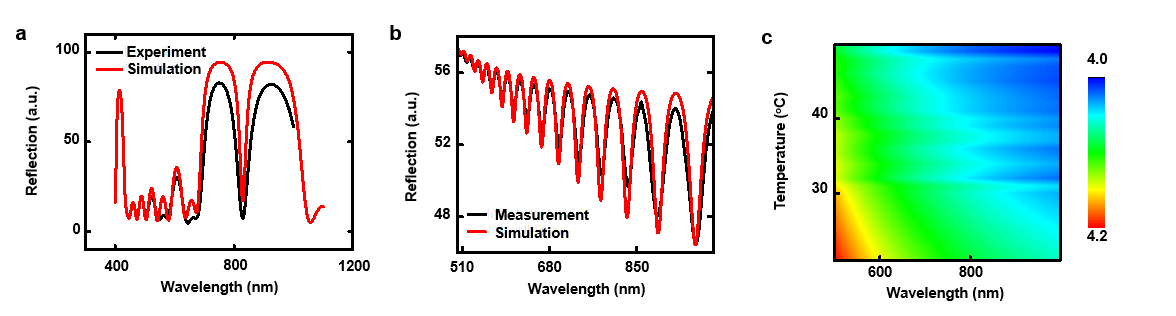


**Supplementary Figure 2.** **(a)** Experimental measurements and simulation results of transmission spectra of the SiO_x_/SiN_y_ multilayer layer at a 0^o^ incidence angle. **(b)** Experimental measurements and simulation results for transmission spectra of a 1.5 µm-thick Si membrane at a 0^o^ incidence angle. **(c)** Experimental measurements of refractive index of a Si membrane as a function of temperature (from 21 ^o^C to 50 ^o^C) and wavelength (from 500 nm to 1000 nm), determined using an ellipsometer. The device consists of a 1.5 µm-thick Si membrane supported on a 10 µm-thick PLGA film.


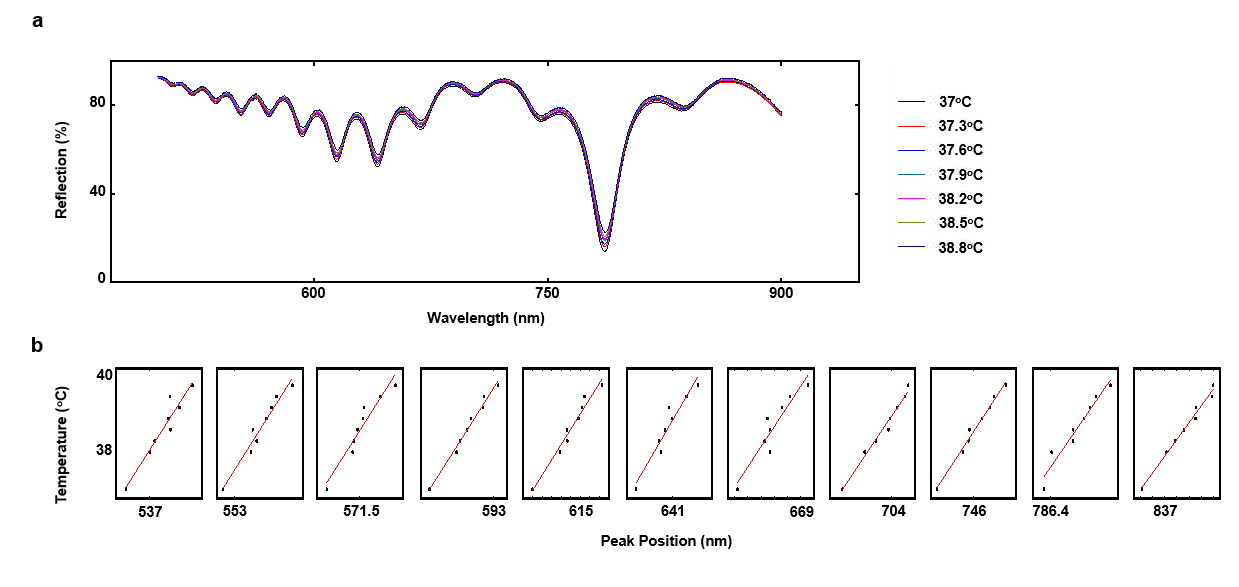


**Supplementary Figure 3.** **(a)** Measured reflection spectra, at 0^o^ incidence angle, of a bioresorbable multilayer photonic cavity in free space as a function of environmental temperature. **(b)** Measured positions of the resonant peaks as a function of temperature. The plot indicates a linear relation between these positions and temperature.


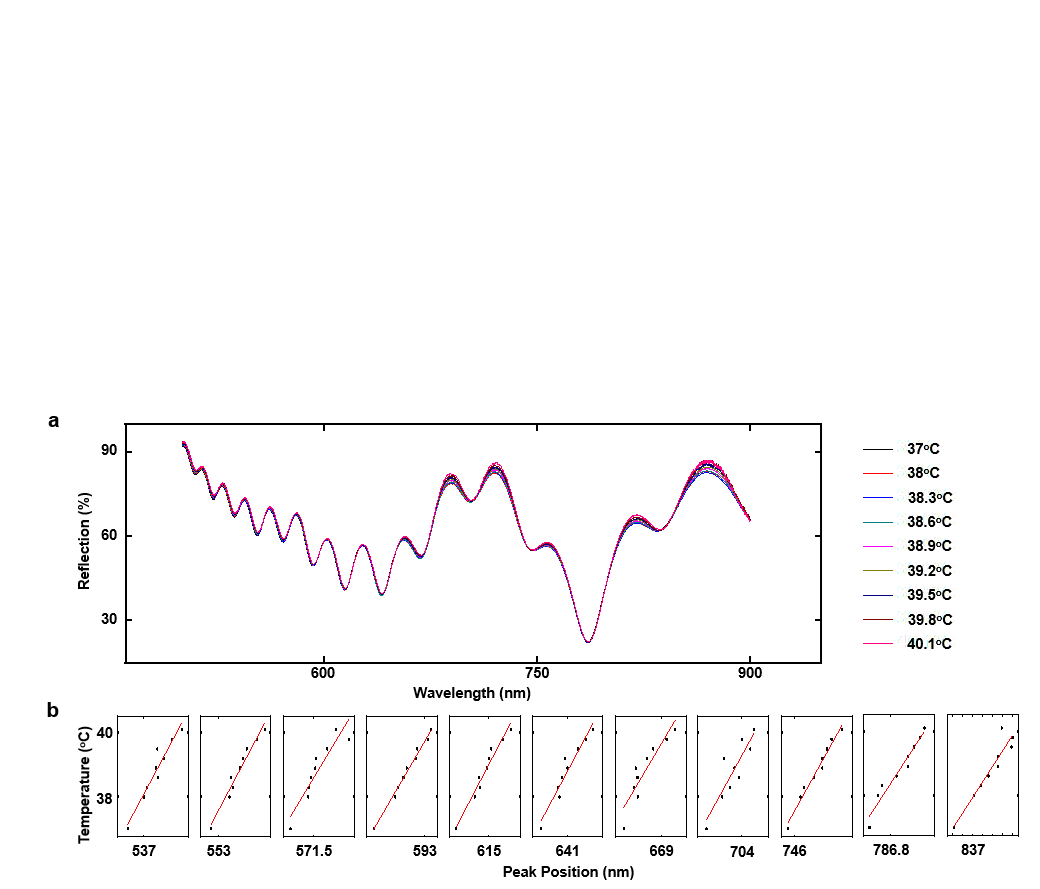


**Supplementary Figure 4.** **(a)** Measured reflection spectra at 17^o^ incidence angle for a bioresorbable multilayer photonic cavity in free space as a function of temperature. **(b)** Measured positions of resonant peaks as a function of temperature. The plot indicates a linear relation between these positions and temperature.


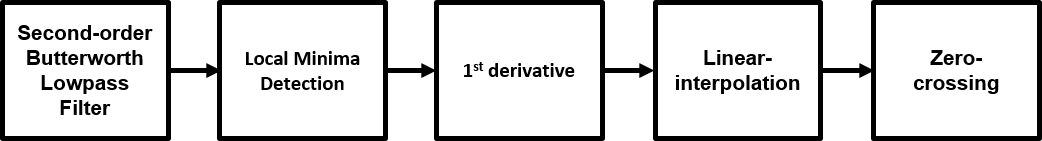


**Supplementary Figure 5.** A flow chart for the denoising algorithm for locating each resonant peak of a measured spectrum. Specifically, a second-order butter-worth lowpass filter with a cutoff coefficient of 5 normalized by frequency resolution was applied to the spectrum once forward and backward for denoising. The local minima of the discrete spectrum were detected with minimum peak distance of 5. After locating each peak F[n], the true peak was estimated by a zero-crossing applied to a linear interpolation between the gradient of spectrum F’[n-1] and F’[n+1]

**
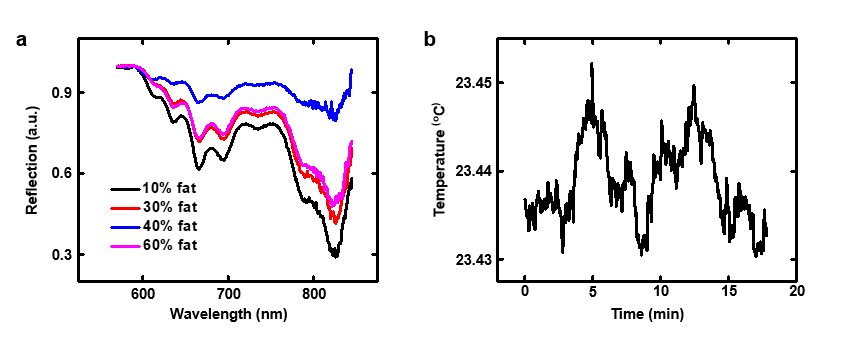
**

**Supplementary Figure 6. (a)** Measured reflection spectra of a bioresorbable multilayer photonic cavity buried underneath 4-mm thick beef slices with various compositions of fat, ranging from 10% to 60%, with the rest of the beef slices mostly composed of muscle tissue. Existence of fat tissue in the beef slices introduces heterogeneity along the optical pathways to the buried photonic cavity, which modulates the resultant reflection spectra and produces noise in measurements. **(b)** Measured temperature of a multilayer photonic cavity as a function of illumination time using a source of white light with irradiance 30 mW/cm2. The measurement is achieved by placing a NTC (Negative Temperature Coefficient) thermistor in close proximity to the multilayer photonic cavity.


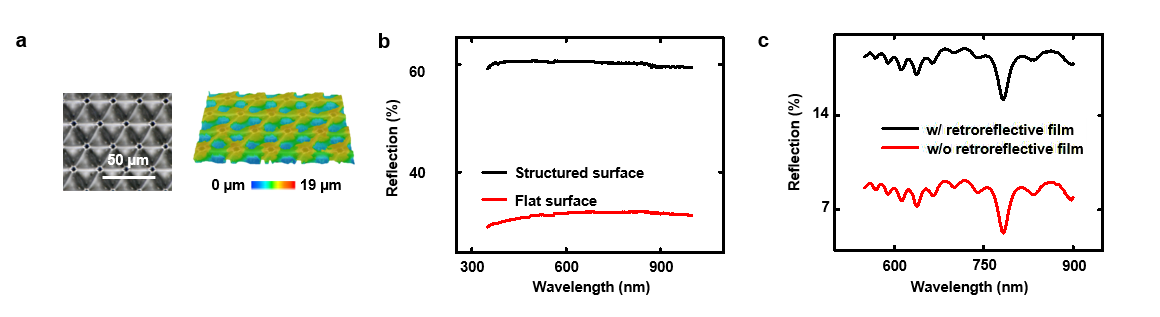


**Supplementary Figure 7.** **(a)** Optical image and 3D optical image of a 10 µm-thick PLGA film with a bottom surface structured with arrays of microscale pyramidal features. **(b)** Reflection spectra of 10 µm-thick PLGA films with a flat surface, and with a bottom surface structured with arrays of microscale corner cubes. **(c)** Reflection spectra of devices with and without retroreflective designs for the PLGA substrates. The devices are embedded in a 5 mm-thick piece of chicken tissue at room temperature (~ 23 ^o^C).


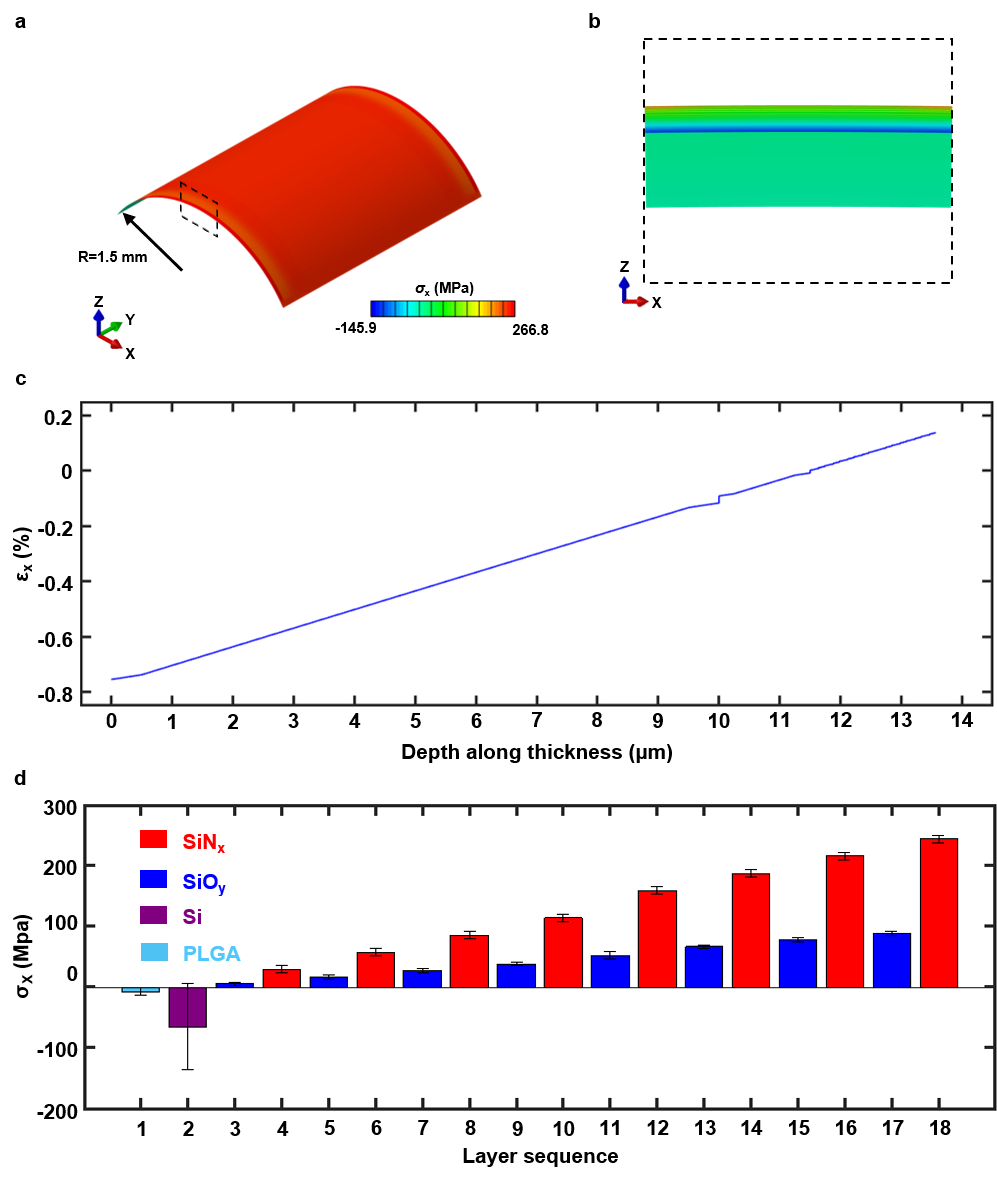


**Supplementary Figure 8. Finite element simulation on the mechanical response of a bioresorbable multilayer photonic cavity structure to bending. (a)** The stress distribution of a device (length x width, 3 mm x 3 mm) during bending to a radius of curvature of 1.5 mm. **(b)** a zoom-in view of the stress distribution at a cross-sectional area (as indicated by the dashed square in (a)) of the device. **(c)** Simulation results on the strain as a function of depth along the thickness of the device (z direction in (b)). The resultant changes in thickness of Si, SiO_x_, and SiN_y_ are less than 0.3 nm, 0.03 nm, and 0.1 nm, respectively. **(d)** The stress exerted on each layer of a multilayer photonic cavity structure (length x width, 6 mm x 6 mm) during bending to a radius of curvature of 1.5 mm.. Error bars denote the stress range in each layer.


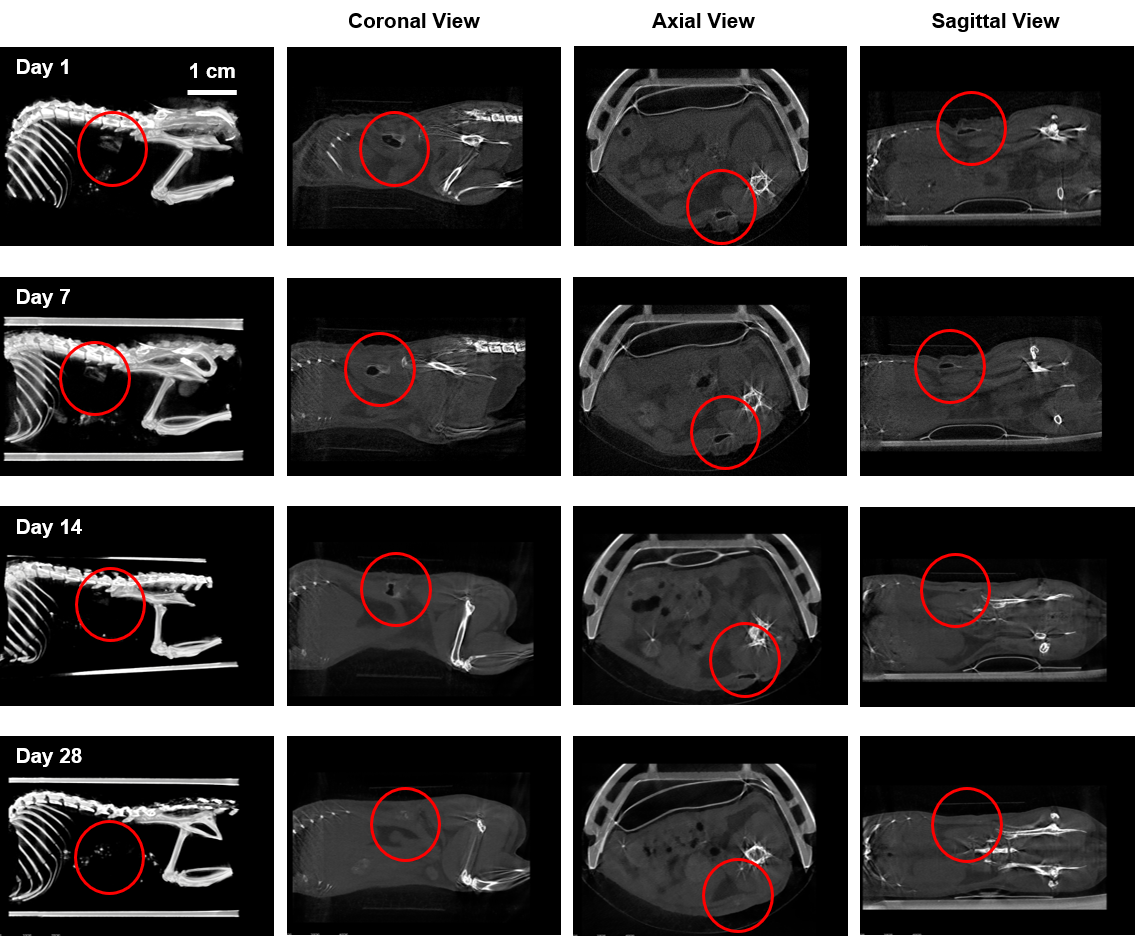


**Supplementary Figure 9.** Computed tomography (CT) images (3D-rendered, coronal, axial, and sagittal views) of mice collected over 2 weeks after implantation of a bioresorbable multilayer photonic cavity, showing gradual disappearance of the device (highlighted by red circle).


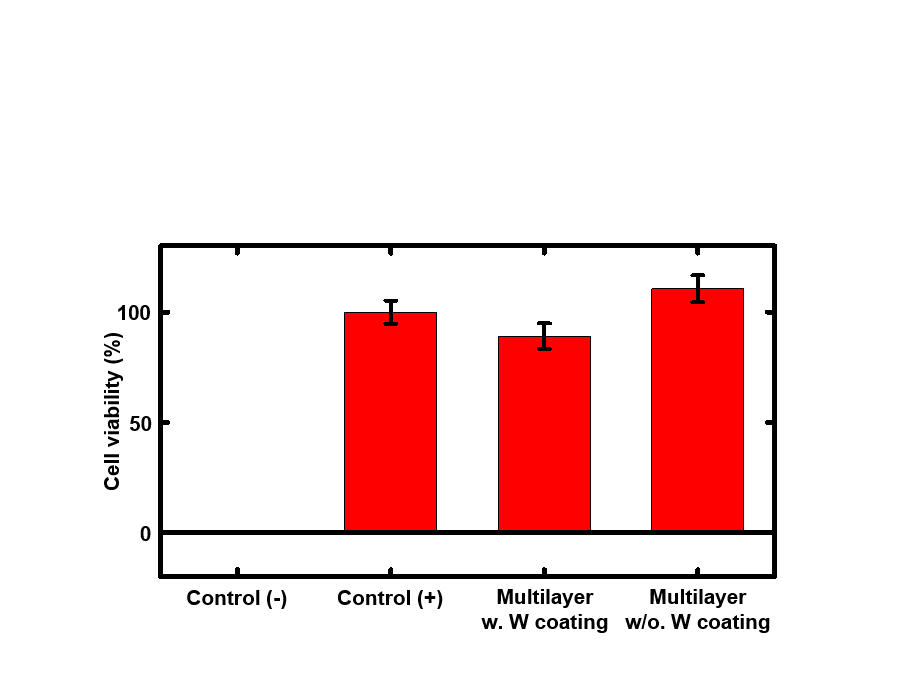


**Supplementary Figure 10.** Cell viability calculated as the fraction of total living cells after conditioning the culture for 24 h.


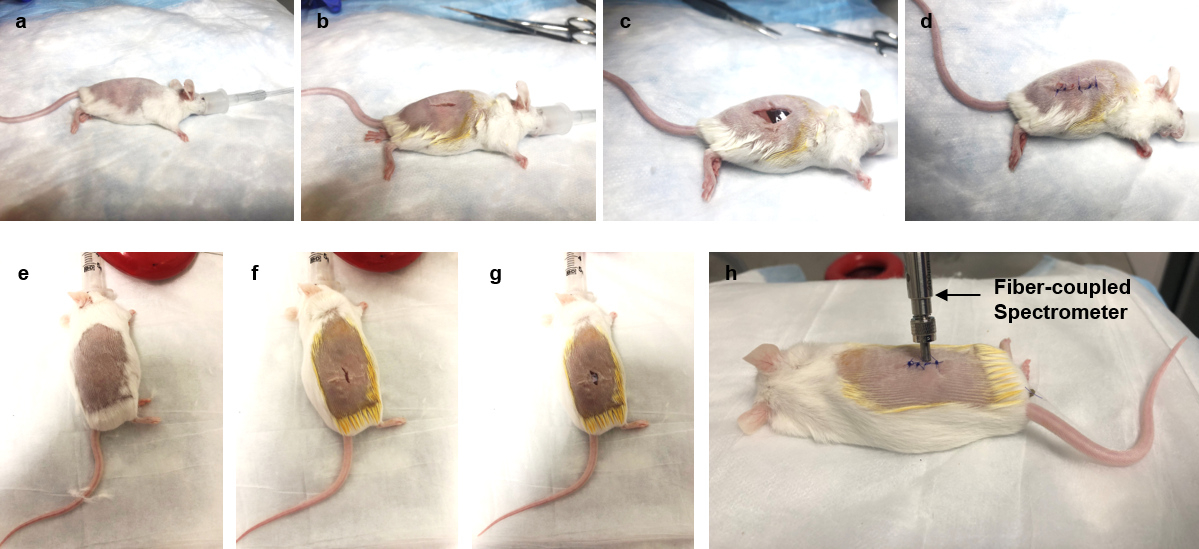


**Supplementary Figure 11. Images of the surgical procedure for implanting a bioresorbable multilayer photonic cavity.** **(a) (e)** Isoflurane vapor anesthetizes a mouse with fur around the surgically removed region. **(b) (f)** Surgical exposure of the subcutaneous region near the thoracic spine. **(c) (g)** Insertion of a biosensor into the surgically exposed region. **(d)** Bioresorbable suturing and gluing close the surgically exposed region. **(h)** Placing a fiber-coupled spectrometer allows for collection of reflection spectra from the device.


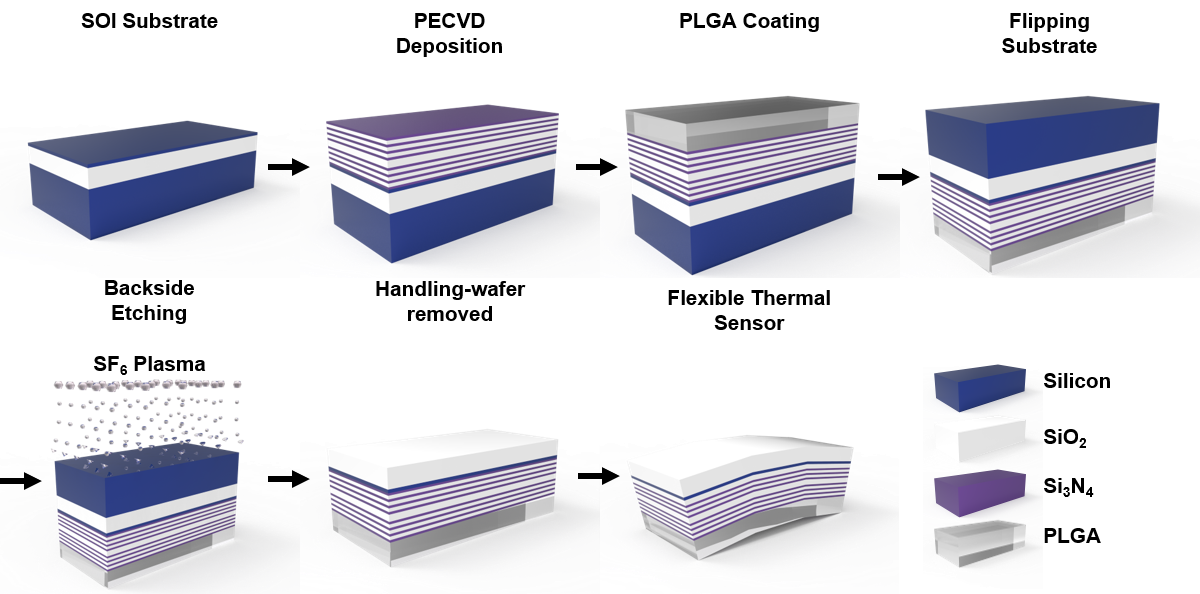


**Supplementary Figure 12.** 3D schematic illustration of key fabrication steps for a bioresorbable multilayer photonic cavity.


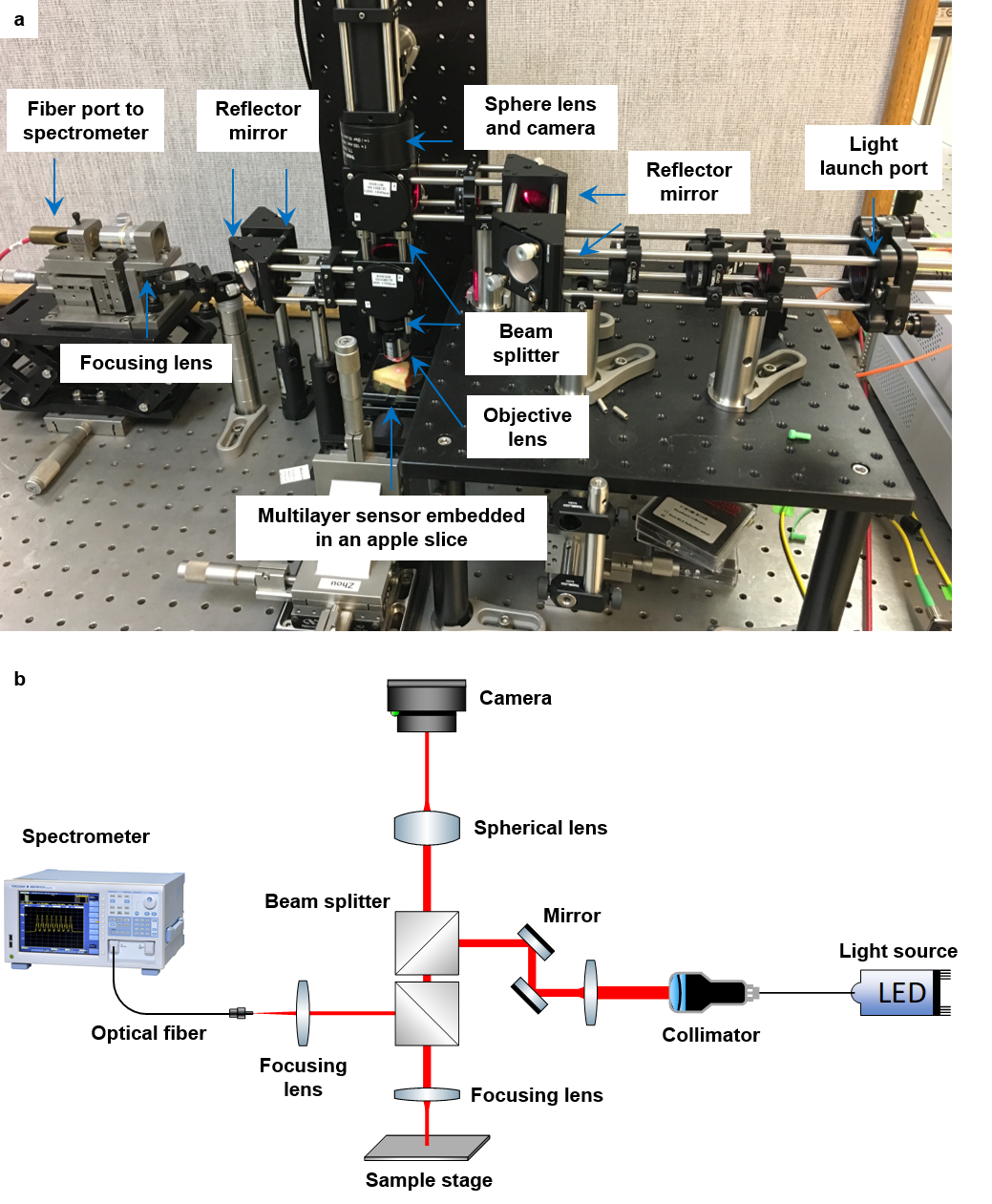


**Supplementary Figure 13. (a)** Experimental setup for optical characterization of the multilayer photonic cavity sensors embedded in an apple slice, in chicken breast tissue and phosphate-buffered saline. **(b)** Schematic illustration of the experimental setup.
